# Developmental Topographical Disorientation With Concurrent Face Recognition Deficit: A Case Report

**DOI:** 10.3389/fpsyt.2021.654071

**Published:** 2021-06-25

**Authors:** Maria Luisa Rusconi, Giulia Fusi, Chiara Stampatori, Angelo Suardi, Chiara Pinardi, Claudia Ambrosi, Tommaso Costa, Flavia Mattioli

**Affiliations:** ^1^Department of Human and Social Sciences, University of Bergamo, Bergamo, Italy; ^2^Department of Clinical and Experimental Sciences, University of Brescia, Brescia, Italy; ^3^Clinical Neuropsychology, ASST Spedali Civili, Brescia, Italy; ^4^Medical Physics Unit, ASST Nord Milano, Milan, Italy; ^5^Neuroradiology Unit, Spedali Civili, Brescia, Italy; ^6^FOCUS Lab, Department of Psychology, University of Turin, Turin, Italy

**Keywords:** developmental topographical disorientation, neurodevelopmental disorders, cognitive map, face recognition, spatial navigation, case report

## Abstract

Developmental topographical disorientation (DTD) has been defined as a developmental deficit in human navigational skills in the absence of congenital or acquired brain damage. We report the case of Lost In Space Again (LISA), a 22-year-old woman with a normal development and no clinical history of neurological or psychiatric diseases, evaluated twice, with an interval of 5 years. The magnetic resonance imaging (MRI) examination did not reveal any morphological alteration, while diffusion tensor imaging (DTI) showed a structural connectivity deficit (a decreased fractional anisotropy—FA) in the parieto–prefrontal and parieto–premotor pathway. The behavioral assessment showed different deficits in spatial and navigational tasks, which seemed to be connected to a poor ability to form a cognitive map of the environment. Moreover, LISA displayed a poor performance in high-level face encoding and retrieval. The aim of this case report is to share new insight about DTD in order to deepen the knowledge of this specific neurodevelopmental disorder. In conclusion, this novel DTD case (1) supports the hypothesis of the existence of different DTD subtypes; (2) sustains the evidence that DTD can co-occur (or not) with deficit in face recognition; and (3) highlights the need for an in-depth examination from both a neurocognitive and behavioral point of view of a possible common developmental defect between the formation of cognitive maps and the recognition of faces that might be in mental imagery skills. Future directions will be also discussed.

## Introduction

In the last years, a newly discovered neurodevelopmental disorder identified as “developmental topographical disorientation” (DTD) has been described. This disorder seems to be widespread (1, 2); however, only few patients affected by DTD have been studied with a comprehensive neuropsychological, behavioral, and neuroimaging assessment. Typically, patients' general intelligence is in the normal range, and they do not report any cognitive complaints except for a really disabling deficit to orient themselves and to find their way in new and/or even in familiar environments since childhood (3–9). They also have no history of neurological or psychiatric disorders and show no brain damage. Moreover, even if the greatest difficulty exhibited by these patients was found in the formation of a cognitive map (2), they have exhibited varying types and degrees of spatial and navigational difficulties [see Fusi et al. (10) for a review] leading researchers to hypothesize the existence of different subtypes of DTD (8). The same emerged for the evaluation of neuroimaging evidence, with patients that showed different patterns of alterations in terms of activations or functional connectivity (FC) (3–6, 8, 11, 12). In addition, interestingly, some of the described DTD cases showed impairment in face recognition tasks (10) or even a clear diagnosis of prosopagnosia (7). The authors have already suggested that a possible common developmental defect could be responsible for this comorbidity.

Here, we present a new DTD case, characterized not only by spatial and navigational deficits but also by impairments in two face recognition tasks. Our aim is to share new insight about DTD by highlighting again the need to provide a new taxonomy and, more specifically, to refocus the discussion about the possible link between navigational deficits and face recognition abilities.

## Case Description

We present the case of a 22-year-old woman, Lost In Space Again (LISA). She had a high school diploma and no previous clinical, neurological, or psychiatric disorders. She was referred for a neuropsychological examination because of her disabling life-long difficulties to orient herself in the environment. The patient has been experiencing difficulties in space orientation since she was a child. She reported difficulties in learning new routes and in mentally representing environments, even of familiar places such as her neighborhood. Moreover, she textually described her difficulties as “a struggle to connect two known places,” or to “retrace a path backward:” she therefore reported some episodes in which she got lost and she had to return to “the starting point” (her home) to properly reach the initial destination. The patient was submitted to a clinical psychological interview, which also provided for the administration of the Cognitive Behavioral Assessment 2.0 [CBA 2.0; (13)] and the Minnesota Multiphasic Personality Inventory-2 [MMPI-2; (14)] from which no psychological disorders have been evidenced. LISA did not report any other cognitive or emotional efforts, except for a slight status of anxiety due to navigational deficits. Then, a first comprehensive assessment was performed and, 5 years later, due to the persistence of the orientation difficulties, the patient returned to our observation and she was submitted to a complementary evaluation (second assessment). We obtained the participant's written informed consent. The study was designed in accordance with the principles of the Declaration of Helsinki and received the approval from the Ethics Committee of the University of Bergamo.

### First Assessment

A magnetic resonance imaging (MRI—a 1.5-T MRI scanner MAGNETOM Avanto, Siemens, Erlangen, Germany) was performed; the examination did not show any morphological alteration. LISA was then submitted to a first standard neuropsychological (NPS) evaluation (see Table 1).

**Table 1:** Results of LISA's first neuropsychological assessment (13 years of education).

**Test**	**Patient's score**	**Cut-off/equivalent score (ES)**
**General intelligence**		
Montreal cognitive assessment (15)	28/30	26/30
WAIS-R (16, 17)^*^		
Digit symbol (WAIS-R)^*^	14	4
Object assembly (WAIS-R)^*^	7	4
Block design (WAIS-R)^*^	9	4
**Imagery abilities**		
Mental rotation test (18)^**^	**6/10**	***M*** **=** **9.05; SD** **=** **1.4; CH:** ***t*** **=** **−2.093;** ***p*** **=** **0.06**
**Reasoning**		
Standard progressive matrices [SPM; (19)]	44/48	75th percentile
**Comprehension**		
Token test (20)	33/36	29
**Verbal memory**		
Digit span (21)	5.5	3.75
Short story recall (22)	15	ES = 4
15 Rey's words (23)		
- Immediate	61.4	ES = 4
- Recall	15	ES = 4
**Visuo-spatial memory**		
Corsi supra span (20)	14.61	ES = 3
Corsi span (21)	4.5	3.5
Rey's complex figure (24)		
Copy	32.5/36	ES = 4
Delayed recall	**9.75**	**ES** **=** **1**
**Attention**		
TMT A (25)	55”	ES = 2
TMT B	177”	ES = 2
TMT B-A	107”	ES = 2
**Divided attention** [TEA; (26)]		
Auditory mode	404 ms	*T* = 50
Visual mode	842 ms	*T* = 48
Number of errors	2	*T* = 47
Number of omission	1	*T* = 50
**Executive functions**		
Clock test [ENB2, (27)]	10/10	8
CET (28)		
- Absolute error score	14	>18
- Bizarreness	4	>4
Phonemic verbal fluency (22)	50	ES = 4
Semantic verbal fluency (22)	47	ES = 4
Stroop test (24)
Time interference	0	ES = 4
Error interference	0	ES = 4
Elithorn's perceptual maze test (20)	21.25	ES = 3
Tower of London (29)		
Total correct score	92	<69
Total problem-solving time	98	–
Total time violation	92	–
Total rule violation	0	–
Wisconsin card sorting test (WCST) (30)		
% errors	14	*T* = 63
% perseverative errors	12	*T* = 55
% not perseverative errors	2	*T* = 75
Benton line orientation test (31)	23/30	15/30
Benton facial recognition test (32)	**39**	**39/54**
**Spatial and navigational abilities**		
Manikin test (33)	32/32	–
Road map test (34)^**^	27/32	*M* = 23; SD = 2.1
Map of Italy (20)	10.5	7.5
VVIQ (35)^**^	74/80	*M* = 64.9; SD = 9.03
VMIQ	120/120	–
TVIC (35)	50/50	–

* *WAIS-R tasks have been selected from the full scale in order to evaluate only the patient's visuo-spatial abilities*.

** *A 20-subject control group matched for age, gender, and education was used for the statistical analysis. CH referred to Crawford and Howell (36) analysis made by SINGLIMS.EXE. M, mean; SD, standard deviation; T, t-score. Performances below or close to the cut-off are in bold*.

LISA was alert and cooperative and not impaired in general intelligence, reasoning, and language, but she showed selective deficits that will be addressed in the Discussion section.

### Second Assessment

**Figure 1:**
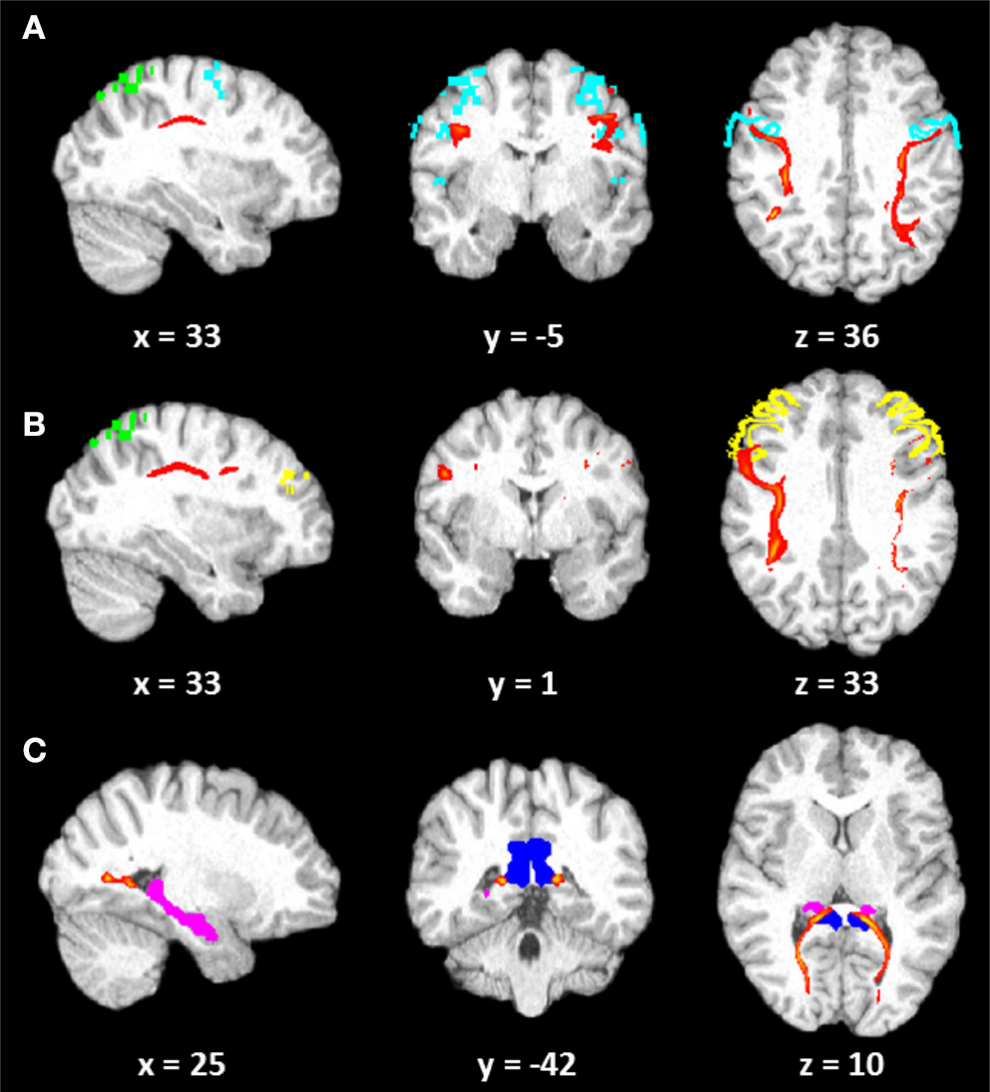
Diffusion tensor imaging (DTI) images. Tracts (red) reconstructed (10% threshold) in LISA: **(A)** connection between Brodmann area (BA) 7 (green) and BA6 (light blue); **(B)** connection between BA7 (green) and BA9 (yellow); **(C)** connection between the posterior cingulum (blue) and the hippocampus (pink). Coordinates are in Montreal Neurological Institute (MNI) space.

Five years later, LISA was submitted to a second complementary evaluation. Her cognitive status remained essentially unchanged (see Supplementary Material), and the psychological measures [i.e., Beck Depression Inventory, BDI—(37); State-Trait Anxiety Inventory, STAI—(38)] were in the normal range. A diffusion tensor imaging (DTI) was then performed with the same scanner of the previous evaluation using the following scanning procedure: 1. DTI (gradient echo EPI) sequence: 2 mm isotropic voxel, 20 encoding directions, 2 avg/dir, effective *b* value of 1,000 s/mm^2^ and 2. high-resolution T1 3D MPRAGE: TR/TE 2,050/2.56 ms, 256 mm FOV, 256 ×256 matrix, and 144 sagittal slices for an effective resolution of 1.0 mm^3^. All data processing was performed using the FMRIB Software Library (FSL) tools (http://www.fmrib.ox.ac.uk/fsl). Source images of each run were corrected for distortions caused by eddy currents and head motion with an affine registration to the first *b* = 0 image using the FMRIB's Linear Image Registration Tool (FLIRT) and Diffusion Toolbox. Next, the two runs were concatenated and then averaged. The *b* = 0 mean image was coregistered to the 3D T1-weighted anatomical image, which was normalized to standardized Montreal Neurological Institute 152 (MNI) space. The fractional anisotropy (FA) maps was calculated using the DTIFIT tool. Probabilistic tractography was run using FSL's BedpostX program, based on a multifiber diffusion model (39) with the aim to explore the visuo-spatial networks proposed by Kravitz et al. (40), investigating the anatomical connection between the Brodmann area (BA) 7 and the BA6 and BA9 and between the posterior cingulate and hippocampus. DTI images are shown in Figure 1.

The mean FA values along each right and left fiber tract were extrapolated from patient's DTI data. A group average template, constructed by Rohlfing et al. (41), was used in order to compare patient's results with only an observatory purpose. LISA showed global decrease of FA with respect to the normal subjects in the parieto–prefrontal and in the parieto–premotor pathways. However, comparable (in the right hemisphere) or greater (in the left hemisphere) FA values were observed in the patient as concerned the parieto-medial-temporal pathway. The decrease of FA related to a parieto–prefrontal pathway, which is supposed to support spatial working memory and top-down executive control of visuo-spatial processing, and to a parieto–premotor pathway, involved in visually guided actions (40), might have an impact on her ability to integrate and manipulate different frameworks of spatial information and to form a complex internal space representation.

LISA then performed ecological spatial and navigational tasks (see Table 2). Specifically, in order to test wayfinding abilities in familiar surroundings, she was submitted to two tasks in which she had to describe 12 paths from her home to other places (*Wayfinding in familiar routes 1*) and 12 paths from one place to another in her town (*Wayfinding in familiar routes 2*). Then, we used two other ecological navigation tests: in the first task, she had to draw a map of her home and of her hometown to test her ability to retrieve cognitive maps of very familiar environments. An adapted version (i.e., related to the city where the patient lived) of the Postcard Test of Palermo et al. (42), named “The Landmark Test,” was also administered to evaluate patient's ability to recognize landmarks (buildings, palaces, and monuments) or views (streets and squares) of known and unknown places and to relocate them on a blank map of the city.

**Table 2:** Results of LISA's ecological spatial and navigational assessment.

**Test**	**LISA scores**	**Controls (C)**
Wayfinding in familiar routes 1 (from home to places in her town)^*^	11/12	–
Wayfinding in familiar routes 2 (from places in her town to home)^*^	3/12	–
Map drawing of her home^*^	Plausible	+
Map drawing of her hometown^*^	Poor	+
Landmark test (adapted postcard test)^**^		
Recognition of hometown landmarks	10/10	*M* = 10; SD = 0
Recognition of European landmarks	10/10	*M* = 10; SD = 0
Hometown landmarks replacement	**5.5/10**	***M*** **=** **9.00; SD** **=** **0.45; CH:** ***t*** **=** –**9.129;** ***p*** **=** **0.001**

* *patient's performance was balanced with the descriptions collected by her mother and friends and it was considered to be exact if it included the correct sequence, direction and turns; + patient's performance was considered by three independent judges and patient's mother*.

** *Our adapted test consisted in 30 postcards of, unknown places (10), known places in European cities (10) and known places in the patient city (10). Initially, she had to recognize all the landmarks and then she had to relocate them on a blank map of the city. Performance was scored as follows: 1 point = a correct response (the name of the landmark/view), 0.5 points = an appropriate response (name of the landmark/view) but in an incorrect position, and 0 point = an incorrect response. A five subject–control group, matched for age (m = 27.2; SD = 5.22) gender, and education (m = 16.2; SD = 1.92), was considered. M, mean; SD, standard deviation. CH referred to Crawford and Howell (36) analysis made by SINGLIMS.EXE. Not sufficient performances are in bold*.

LISA was finally submitted to a battery of online tests assessing different cognitive skills relevant to DTD, which includes both spatial and face recognition tasks [see www.gettinglost.ca and Iaria and Barton (1) for a detailed description], and to the “Plastic City test,” which was used as a small-scale unfamiliar but more ecological environment [see Rusconi et al. (43) for a detailed description]. LISA's performances are shown in Table 3.

**Table 3:** LISA's results in *on-line T* and *Plastic City test*.

**Test**	**LISA scores**	**Controls (C)**
**On-line T subtest**		
Object recognition	10/10	–
Face identity recognition	**6/10**	–
Face expression recognition	9/10	–
Landmark recognition	8/10	–
Heading orientation	8/10	–
Left/right orientation	**Not solved**	–
Path reversed	**Not solved**	–
Cognitive map formation	**Not solved**	–
Cognitive map use	**5/10**	–
Plastic City test^*^		
Route learning forward test (errors)	0	*M* = 0.42; SD = 0.67; CH: *t* = −0.602; *p* = n.s.
Route learning forward test (time)	50.00 s	*M* = 31.42; SD = 19.28; CH: *t* = 0.926; *p* = n.s.
Route learning backward test (errors)	0	*M* = 0.08; SD = 0.29; CH: *t* = −0.265; *p* = n.s.
Route learning backward (time)	48.00 s	*M* = 25.08; SD = 14.58; CH: *t* = 1.510; *p* = n.s.
Free recall landmark	**6.00**	***M*** **=** **15.92; SD** **=** **2.64; CH:** ***t*** **=** **−3.610;** ***p*** **=** **0.004**
City landmark replacement (CLR)	**2.00**	***M*** **=** **4.75; SD** **=** **0.34; CH:** ***t*** **=** **−7.771;** ***p*** **<** **0.001**
Map drawing	39.00	*M* = 35.54; SD = 6.77; CH: *t* = 0.491; *p* = n.s.
Landmark photo recognition	7.00	*M* = 7.58; SD = 1.31; CH: *t* = −0.425; *p* = n.s.
Map replacement	5.00	*M* = 6.58; SD = 1.31; CH: *t* = −1.159; *p* = n.s.
Recall replacement on map	2.00	*M* = 4.25; SD = 1.22; CH: *t* = −1.772; *p* = n.s.
Route planning	3.50	*M* = 4.04; SD = 0.72; CH: *t* = −0.721; *p* = n.s.
Short route planning	3.50	*M* = 4.58; SD = 1.47; CH: *t* = −0.706; *p* = n.s.
Route learning forward test 2 (errors)	0	*M* = 0; SD = 0
Route learning forward test 2 (time)	**43.00 s**	***M*** **=** **20.25; SD** **=** **8.32; CH:** ***t*** **=** **2.627;** ***p*** **=** **0.024**

*M, mean; SD, standard deviation*.

* *LISA's performances were compared to that of a 12-subject control group (C), matched for age (m = 27.25, SD = 2.67), gender, and education level (m = 17.25, SD = 1.36), by means of the analysis developed by Crawford and Howell (36) (CH) and Crawford and Garthwaite (44) using the computer program SINGLIMS.EXE. Not sufficient performances are in bold*.

## Discussion

We described a new DTD case, LISA, whose assessments revealed a complex clinical picture and presented some significant elements that may be considered for future studies. The NPS evaluation showed a normal general cognitive profile and normal abilities to recognize objects, landmarks, and face expressions and to recall directional information from landmarks in the *on-line T*. Her difficulties seemed to begin when she had to form, have access, or manipulate a cognitive map in order to reach a destination. Indeed, her performances were altered in the formation and use of a cognitive map and in the *path reversed subtest*, which requires to form a cognitive map and to perform a manipulation to reverse a route from the final position back to the starting point. These difficulties were confirmed also in the *landmark test* in which, despite good landmark recognition skills, LISA showed significant difficulties in the relocation of these landmarks on a blank map of her city; the same happened with the small-scale ecological task (i.e., Plastic City), in particular in the *city landmark replacement subtest*. Moreover, her performance was good when she was required to draw a relatively simple or overlearned cognitive map (i.e., her home) but was poor in the reproduction of a more complex environment such as her neighborhood. Again, when she had to describe some paths giving verbal information, her performance was good only when the starting point was her home (i.e., overlearned path). Conversely, a clear impairment was observed when the starting point was in any other place in her hometown, even when the target point was her home, as if she was able to access an overlearned cognitive path/map, but she was unable to manipulate and rotate this information in order to complete the second task. All this evidence (confirmed also by the altered performance in the Rey recall task) seems to suggest that LISA was actually unable to form quickly a complex internal representation of the environment that would allow her to access the information about landmarks and their mutual metric distances; she was able to form a representation only after multiple presentation of the same environment (i.e., overlearned). According to this, but contrary to the findings of the study performed by Burles and Iaria (2), LISA showed a deficit in the *mental rotation test*. The capability to perform mental rotations has already been linked to the ability to form cognitive maps: some authors, indeed, evidenced how this imagery ability could help subjects to build spatial relationships between landmarks (42). This can be seen as a first indication that DTD subjects can show different patterns of imagery and spatial abilities and therefore that different subtypes of DTD can be hypothesized (8).

Another interesting aspect, about the possible existence of different DTD subtypes, is that LISA revealed also a poor performance in high-level face recognition abilities: only some of the DTD cases showed this deficit [(4, 6, 9, 11); see (10) for a review]. She seemed able to recognize face expression, which required low-level abilities, but showed significant difficulties in the face identity recognition subtest and in the *Benton Face Recognition Test*. Grounding on the model proposed by Bruce and Young (45), LISA might have a specific deficit in the structural encoding of faces, which concerns the ability to create a complex mental representation of a pattern of invariant face characteristics against a spared ability to recognize face expression, which instead concerns changeable face characteristics (46). It is also worth noting that previous studies showed that subjects affected by both developmental prosopagnosia (DP) and acquired prosopagnosia (AP) showed concurrent topographical deficit (47, 48). Indeed, even if the comorbidity between DP and navigational difficulties or DTD is not yet known, some evidence does suggest that many subjects might experience both types of difficulties [e.g., Corrow et al. (49); Klargaard et al. (47); Piccardi et al. (7)]. It was already hypothesized that this relationship is possibly driven, on the one hand, by the fact that visual processing of faces and places, even if they are distinct enough to be clearly dissociable, are both ventral stream processes (2); however, this discussion has not been continued to date. For example, also the well-known role of the ventral stream in the processing of far space and allocentric representations and its connection with the dorsal stream (50) might be considered more carefully in future studies. Indeed, subjects with DTD seem to be able, like LISA, to recognize spatial landmarks (a deficit usually found in patients with lesions in these areas), but they seem to have higher-level difficulties that could be explained by alterations in the structural or functional connections with other areas belonging to the spatial network. On the other hand, other authors have evidenced the possible role of the hippocampus that is implicated both in the encoding of new faces (51) and of verbal and spatial information (52, 53), so much so that lesions to this structure or surrounding areas can result in higher-order impairments of both face recognition (54) and/or to the inability to encode and retrieve spatial information about newly learned routes (55). This is in line with the few neuroimaging findings, which have revealed a lower hippocampal and retrosplenial cortex activations during map formation in some DTD subjects (3, 5).

Moreover, from a cognitive point of view, Bate et al. (48) suggested that there could be also an overlapping of cognitive processes involved in both cognitive map formation and face recognition: one of those might be visual imagery skills. Accordingly, we hypothesized that the alteration of mental imagery ability found in LISA might be the possible link between her spatial and face recognition alterations. Consequent to this alteration, LISA was indeed not able to encode, form, and manipulate complex mental images or representations, that could be a face, a scene (environment), or a complex navigational pattern in which the construction of an integrated mental image (cognitive map) was required. It is now clear that some overlaps between the formation of cognitive maps and face recognition abilities do exist and that a possible common developmental defect can be observed; according to this case, mental imagery could then be a prerequisite for the normal development of human navigational skills; however, further studies are needed to investigate this from both cognitive and neural perspectives.

It should be noted that our case presents some limitations: for example, a more in-depth psychological and psychiatric evaluation that could consider the presence of other neurodevelopmental pathologies in comorbidity as well as functional imaging data that could give additional significant information. However, this novel DTD case has certainly the value to bring the attention back to the possible presence of face recognition disorders in patients with DTD [and on the comorbidities with prosopagnosia, see Piccardi et al. (7)] and to highlight again how different forms of this disorder may exist, leading to the hypothesis of the possible existence of a specific taxonomy for individuals with DTD. Future studies should therefore investigate DTD patients both with psychological, psychiatric, behavioral, and functional neuroimaging measures to establish the cognitive and neural profile of different subtypes of DTD; observatory DTI results seemed to suggest also that alterations in the connection between brain areas involved in the human navigational network might be further explored. Comprehensive batteries not only should include specific spatial and navigational tasks but could also investigate the domain of face processing and recognition, as suggested by Burles and Iaria (2), by demanding more ecological stimuli (e.g., short movies, people embedded in spatial and/or social contexts, etc.) in order to deepen our knowledge about the link between these two disorders. Finally, for the future, it could be interesting to evaluate the processing not only of spatial but also of temporal orientation, given the overlapping of processing (i.e., mental cognitive maps) in these domains [see (56)].

## Conclusions

Spatial navigation is a complex cognitive skill that plays a key role for the proper functioning of daily activities, allowing individuals to navigate in the environment. Alterations in this capacity, such as those demonstrated in children and adolescents with this specific neurodevelopmental disorder (i.e., DTD), can have adverse developmental consequences and even a reduction in the overall quality of life. Different DTD subtypes seem to exist, and therefore, comprehensive psychological, cognitive, behavioral, and neuroimaging assessments are needed to study in detail the development of different cognitive skills in these patients. This case report indeed suggests that not only spatial ability results to be altered in DTD patients but also concurrent spatial and face recognition deficits can be observed, at least in some cases, and that an alteration in mental imagery skills might represent the developmental defect underlying the two altered abilities. Finally, a better understanding of a specific patient's orientation and face processing alterations could be also fundamental to plan *ad hoc* rehabilitation programs.

## Data Availability Statement

The raw data supporting the conclusions of this article will be made available by the authors, without undue reservation.

## Ethics Statement

The studies involving human participants were reviewed and approved by University of Bergamo. The patients/participants provided their written informed consent to participate in this study. Written informed consent was obtained from the individual(s) for the publication of any potentially identifiable images or data included in this article.

## Author Contributions

MLR, AS, FM, and CS designed the experimental procedures. AS administered the tests batteries. CP, CA, and TC worked on the neuroradiological examinations. GF and MLR wrote the article. All authors contributed to the article and approved the submitted version.

## Conflict of Interest

The authors declare that the research was conducted in the absence of any commercial or financial relationships that could be construed as a potential conflict of interest.
