# Supplementary material for: Developmental Topographical Disorientation With Concurrent Face Recognition Deficit: A Case Report
Source: Front Psychiatry. 2021 Jun 25;12:654071. doi: 10.3389/fpsyt.2021.654071 (PMC8267524; doi:10.3389/fpsyt.2021.654071)
Supplement: Supplementary file 1 [file Table_1.DOCX]

Supplementary Material

| **Test** | **Patient’s score** | **Cut-off/ Equivalent Score (ES)** |
| --- | --- | --- |
| **General Intelligence** | | |
| Montreal Cognitive Assessment (Santangelo et al, 2014) | 28/30 | 25.7 |
| **Imagery abilities** | | |
| Mental Rotation Test (Grossi, 1991)* | **6/10** | **M=9.05; SD=1.4; CH: t= -2.093; p= 0.06.** |
| **Reasoning** | | |
| Standard Progressive Matrices (SPM) Raven (Raven, 2008) | 44/48 | 75%ile |
| **Verbal Memory** | | |
| Digit Span (Orsini et al, 1987) | 6 | 3.75 |
| Short Story Recall (Novelli et al., 1986) | 15 | ES= 4 |
| 15 Rey’s Words (Carlesimo et al., 1996) |  |  |
| - Immediate | 63 | ES= 4 |
| - Recall | 15 | ES= 4 |
| **Visuo-Spatial Memory** | | |
| Corsi Supra Span (Spinnler and Tognoni, 1987) | 15 | ES=3 |
| Corsi Span (Orsini et al. 1987) | 4.5 | 3.5 |
| Rey’s complex figure (Caffarra et al, 2002) |  |  |
| -Copy | 31.4/36 | ES= 4 |
| -Delayed Recall | **5.75** | **ES=0** |
| **Attention** | | |
| TMT A (Giovagnoli et al., 1996) | 50” | ES=3 |
| TMT B | 177” | ES= 2 |
| TMT B-A | 126” | ES= 3 |

| **Test** | **Patient’s score** | | **Cut-off/ Equivalent Score (ES)** |
| --- | --- | --- | --- |
| **Executive functions** | | | |
| Clock Test (ENB2, Mondini et al. 2002) | | 10/10 | 8 |
| Phonemic verbal fluency (Novelli et al., 1986) | | 51 | ES = 4 |
| Semantic verbal fluency (Novelli et al., 1986) | | 48 | ES = 4 |
| Verbal Judgment Test (Spinnler and Tognoni, 1987) | | 47.25 | ES= 3 |
| Stroop test (Caffara et al., 2002) | |  |  |
| Time interference | | 0 | ES= 4 |
| Error interference | | 0 | ES= 4 |
| Elithorn’s perceptual Maze Test (Spinnler and Tognoni, 1987) | | 2311 | ES= 3 |
| Tower of London (Culberston et al, 2005) | |  |  |
| Total correct score | | 94 | <69 |
| Total problem solving time | | 98 | - |
| Total time violation | | 92 | - |
| Total rule violation | | 0 | - |
| Wisconsin Card Sorting Test (WCST) (Heaton, 1981) | |  |  |
| % errors | | 13 | T= 55 |
| % perseverative errors | | 8 | T= 50 |
| % not perseverative errors | | 1 | T= 75 |
| **Line orientation and face recognition** | | | |
| Benton Line Orientation Test (Benton et al. 1978) | | 25/30 | 15/30 |
| Benton Facial Recognition Test (Benton et al. 1983) | | **38** | **39/54** |

# Supplementary Table. LISA’s second neuropsychological assessment that included tests for use with Italian-speaking subjects.* A 12-subject control Group matched for age and education was used for the statistical analysis; CH referred to Crawford and Howell (1998) analysis made by SINGLIMS.EXE. es= equivalent score; M= mean; SD= standard deviation. Performances below or close to the cut-off are in bold.
